# Rethinking the fight against pig-related human salmonellosis in the European union

**DOI:** 10.1186/s40813-025-00460-7

**Published:** 2025-10-21

**Authors:** Raúl C. Mainar-Jaime, A. Casanova-Higes, María Bernad-Roche, Juan P. Vico, S. Andrés-Barranco

**Affiliations:** 1 Departamento de Patología Animal, Facultad de Veterinaria, Instituto Agroalimentario de Aragón-IA2, Universidad de Zaragoza-CITA, Zaragoza, 50013 Spain; 2 Pig and Poultry Development Department, The Irish Food and Agriculture Authority, Moorepark, Fermoy, Co. Cork P61 C996, Teagasc, Ireland; 3 Facultad de Ciencias Agropecuarias, IRNASUS-CONICET-Universidad Católica de Córdoba, Universidad Católica de Córdoba, Córdoba, 5000 Argentina; 4 Departamento de Ciencia Animal, Centro de Investigación y Tecnología Agroalimentaria de Aragón, Instituto Agroalimentario de Aragón-IA2, CITA-Universidad de Zaragoza, Zaragoza, 50059 Spain

**Keywords:** Control programs, Foodborne zoonoses, Prediction, *Salmonella*, Slaughter, Swine

## Abstract

The prevalence of human salmonellosis associated with pork products remains a significant concern for public health authorities within the European Union. Despite the implementation of national programs in some member states with the objective of controlling the infection of *Salmonella* in farms, the proportion of human cases involving swine-associated *Salmonella* serotypes has remained constant in recent years, and the majority of these programs were either discontinued or reduced to biosecurity guidance. This article discusses the reasons for the lack of success of these programs, including the focus on the growing-finishing period without consideration of earlier stages of production, the structure of the pig sector, the limited and unrepresentative sampling carried out in the programs, and the use of imperfect serological tests, which have likely resulted in biased estimates of the true health status of the herds. A potential comprehensive approach is proposed, based on predicting the risk of *Salmonella* shedding prior to the arrival of pigs at the slaughter. This knowledge would be combined with the administration of on-farm additives (i.e. organic acids, bacteriophages) during the days prior to slaughter. It would help to reduce shedding in those batches with a high risk of shedding and decrease slaughter environmental contamination. Furthermore, this approach would contribute to obtain more accurate information regarding the *Salmonella* status of the pig farms.

## Introduction

*Salmonella enterica* has been infecting humans in western Eurasia for over 5,000 years [1]. Human-adapted serotypes (i.e., Typhi and Paratyphi) are the most virulent serotypes for humans, causing the so-called typhoid fever. This disease claims millions of cases and 200,000 deaths annually, mostly in low- and middle-income countries where poor sanitation and deficient hygiene infrastructure persist [2]. In high-income countries, however, *Salmonella* has found its way through the non-typhoidal *Salmonella* (NTS) serovars. Infection with these non-host-adapted serovars typically results in self-limiting diarrheal disease with a low case fatality rate.

In the EU, 77,486 cases were officially recorded in 2023 [3], but the actual number is likely to be much higher and could cost up to €3 billion per year, with significant costs arising from hospitalizations, productivity losses, and public health interventions [4–6]. Globally, NTS may cause over 95 million of cases and more than 50,000 deaths [7], with increasing prevalence in developed regions such as the EU, and in many developing countries [3, 8]. These trends are associated with the widespread and emergence of new virulent serotypes (i.e. the monophasic *S*. Typhimurium) as well as of multidrug-resistant and invasive strains of NTS [9–11]. This has prompted the World Health Organization (WHO) to prioritize NTS serovars within the list of pathogens that could trigger future pandemics [12].

Most NTS human infections are associated with contaminated foods of animal or plant origin, resulting directly from infected animals or indirectly contaminated by them [13]. Specific serovars, such as *S*. Typhimurium and *S*. Enteritidis, have been identified as frequently associated with these animal/plant food sources [13]. Therefore, in numerous countries, initiatives have been implemented to reduce the prevalence of infection in food-producing animals, particularly poultry, which is considered the main source of *S*. Enteritidis, the top NTS infecting humans [14]. The control of *Salmonella* in poultry, largely through targeted interventions against it (e.g., vaccination) and the implementation of comprehensive farm-to-fork strategies, has been effective in reducing the incidence of human salmonellosis by this serotype in the EU [15]. However, an increasing trend in human cases of *S*. Typhimurium and its monophasic variant has been observed in recent years, which seems to be associated with pigs [16, 17].

Recent genomic studies indicate that the development of intensive swine production in the EU and the US over the last century, together with the globalization of trade and transportation from these regions, have been pivotal in the global emergence and spread of pig-related *Salmonella* [18]. The modern swine industry has undergone a period of gradual evolution, characterized by a series of incremental improvements in productivity and the steady consolidation of smaller farms into much larger herds, increasing pig population densities and, thus, the potential for some pathogen transmission [19]. Similarly, improved travel and transportation have enabled a significant expansion in the export and import of live pigs (e.g., in 2022 the EU imported and exported 29,054,656 and 34,954,421 live pigs, respectively; 20), contributing to the dissemination of pathogens across different world regions.

It is now widely accepted that pigs and their products represent a significant source of NTS infections in humans, with serotypes such as *S*. Typhimurium, its monophasic variant (*S*. 1,4,[5],12:i:-), *S*. Derby, and *S*. Rissen being particularly problematic [13, 16]. In the EU, the proportion of human salmonellosis cases attributable to pigs, including pork and pork products, was estimated to be approximately 30%, although there were notable regional variations between Southern (43.6%) and Northern EU member states (10.6%) [21, 22]. In other developed countries such as the US, up to 12.1% of the *Salmonella* outbreaks were attributed to pork [23] and, in some regions of Australia, the proportion of cases attributed to pork increased from 20% in the period 2009–2016 to 40% in 2017–2019. This increase was likely associated with the emergence of the monophasic variant of *S*. Typhimurium [24].

**Fig. 1:**
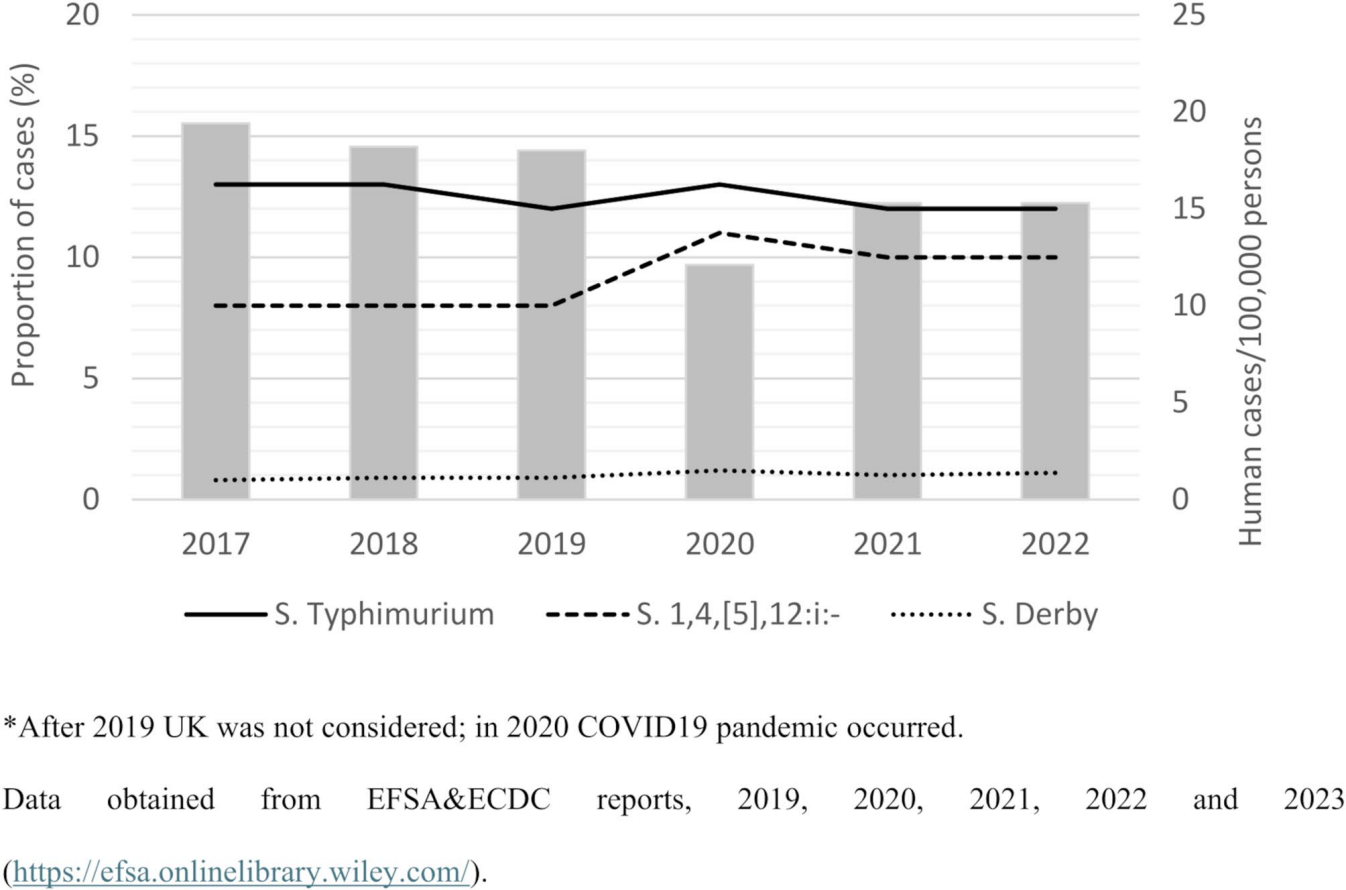
Proportion of major swine-associated serotypes infecting humans in the EU during the period 2017–2022.*

In general, the proportion of cases of human salmonellosis involving swine-associated serotypes (*S*. Typhimurium, *S*. 1,4,[5],12:i:-, and *S*. Derby) has remained fairly constant in the EU during the last years (Fig. 1), suggesting that the efforts carried out to limit the spread of *Salmonella* from pigs to humans have not been successful. It is therefore apparent that new strategies need to be implemented to limit the global spread of NTS from pigs to humans, particularly from areas with a significant pig industry such as the EU. In this article, we discuss possible reasons for the lack of success of most national control programs (NCPs) within the EU and propose new approaches to this problem.

### Control programs against pig salmonellosis in the EU

Following the successful implementation of NCPs targeting salmonellosis in fowls (laying hens, broilers, and turkeys) across all EU Member States (MS) in 2005, according to the framework set by the European Food Safety Authority (EFSA) and EU Regulation No. 2160/2003 [15], attention turned to pigs. It was hypothesized that a reduction in the prevalence of *Salmonella*-infected pigs would result in a decrease in the incidence of human cases associated with pork.

To determine the need for initiating these NCPs, a series of EU-wide baseline surveys were first conducted to assess the prevalence of *Salmonella* in pigs at various stages of production, from breeding to slaughter [25, 26]. These surveys provided crucial insights into the extent of *Salmonella* prevalence in breeding (average of 28.7%) and production (33.3%) holdings in the EU, as well as the prevalence of infection in pigs at slaughter (10.3%) and carcass contamination (8.3%). The results also revealed considerable variability in *Salmonella* prevalence among the MS, ranging from 0 to 64% in breeding holdings and from 0 to 29% in slaughtered pigs. The data also highlighted the dominance of specific serovars, such as *Salmonella* Typhimurium and *Salmonella* Derby, both commonly associated with human infections. These baseline figures should have served as essential data for setting reduction targets and assessing the effectiveness of control programs aimed at curbing *Salmonella* transmission in pig populations within the EU.

Despite the general high levels of *Salmonella* prevalence in pig herds, and the fact that this species was considered the second most important source of human salmonellosis, no further action was taken at farm level in the EU. A comprehensive cost-benefit analysis suggested that there would be no positive economic benefit from setting targets to reduce *Salmonella* in slaughter pigs [27]. Therefore, the decision of whether or not to implement a NCP for salmonellosis in pigs was left to the discretion of each MS, but only a limited number of them decided to do so. These countries were either major producers of swine or demonstrated a high level of commitment to the control of this infection in food-producing animals.

The first comprehensive *Salmonella* NCP (encompassing the entire production chain) for pigs in Europe was already established in Sweden in the 1960s [28]. This was followed by the implementation of comprehensive NCPs in Norway, Finland, and Denmark in 1995 [29]. All of them, except the Danish program, focused on eradication and had bacteriological testing as the cornerstone of their programs. Denmark focused its NCP on *Salmonella* control and relied on both bacteriological and serological analyses [30]. In Norway and Finland the prevalence at the farm level was initially relatively low and strict measures were enforced when *Salmonella* was detected. However, in Denmark, presenting a much larger pig population and higher *Salmonella* prevalence, actions at farm level were less restrictive [31].

Following the success of the Scandinavian action plans, and in line with the EU regulation, new NCPs followed suit in other MS: Germany and the United Kingdom in 2002, Ireland in 2003, the Netherlands in 2005, and Belgium in 2007 (Table 1). In general, these programs were modeled on the Danish approach, that is, focusing on control but using only serology for the surveillance program, which was carried out on a relatively small number of pigs per slaughter batch (from 12 to 72 per year, depending on the country) [30]. Thus, a weighted mean seroprevalence was calculated based on the most recent serological samplings on the farm, and the herds were subsequently classified into three distinct risk groups: low-risk (I), medium-risk (II), and high-risk (III) herds. Category III herds were required to implement specific on-farm measures aimed at reducing their exposure to *Salmonella* and, consequently, their *Salmonella* seroprevalence. While penalties were not typically imposed in most countries, in some cases some potential incentives (i.e. obtaining pork quality labels) were offered to farmers through certification programs. Examples of these certification programs include Qualität und Sicherheit (QS) in Germany [32], British Quality Assured Pork (BQAP) in the United Kingdom [33], Bord Bía Quality Assurance Scheme in Ireland [34], and IKB Nederland varkens in the Netherlands [35].

Estonia was the last MS to implement a mandatory NCP in 2013, but with a different approach. The Estonian NCP is based on the detection of specific serotypes, namely, *S*. Enteritidis, *S*. Typhimurium, *S*. Hadar, *S*. Infantis, *S*. Virchow, *S*. Choleraesuis, *S*. Derby, and *S*. Newport, in pig farms, slaughterhouses, and processing plants [36, 37]. One-fifth of the farms are tested each year, including those that have tested positive for *Salmonella* in previous years and new farms that have not been previously tested. Restrictions are imposed if a farm tests positive.

The Danish NCP came to an end in January 2025. This decision was based on three key factors: the prevalence of *Salmonella* in farms has remained stable in recent years, the prevalence in pork carcasses has remained around 1%, and the number of people becoming ill from consuming Danish pork has also remained low. As a result, the responsibility for ensuring a low prevalence of *Salmonella* in Danish pork will lie solely with the slaughterhouses. (https://svineproduktion.dk/aktuelt/nyheder/2024/10/041024_Salmonella_ophoer). However, the approach taken by most of those countries that had opted for the control of the infection based on serological results from finishing pigs did not yield the expected results. Neither a significant decline in the prevalence of salmonellosis in pigs nor a reduction in human cases related to pork consumption was achieved. Consequently, most programs were discontinued [30].

**Table 1:** Simplified scheme of EU countries that have implemented National control programs against *Salmonella* in pigs and an overview of their results

Country (start year)	Key elements	Results	Success factors / Challenges
**Sweden (1960)** [28]	- Mandatory - Eradication - Emphasis on herd health and biosecurity - Regular bacteriological testing of all farms and slaughtered pigs - Special guarantees for imported meat	Pig herd prevalence < 1% (based on lymph nodes and carcass bacteriology); stringent control measures, including vaccination, have led to almost negligible rates of *Salmonella*	Strong biosecurity measures and effective control at slaughter
**Norway (1995)** [38, 39]	- Mandatory - Eradication - Extensive bacteriological sampling of breeding herds and slaughtered pigs - Focus on low *Salmonella* prevalence through strict biosecurity	Pig herd prevalence ≈ 0.03% (based on lymph nodes and carcass bacteriology)	Extremely low prevalence compared to other EU countries
**Finland (1960)** [40, 41]	- Mandatory - Eradication - Biosecurity, bacteriological surveillance (breeding herds and slaughtered pigs) - Special guarantees for imported meat	Low prevalence at slaughter < 1% (based on lymph nodes and carcass bacteriology) due to extensive biosecurity practices and strict monitoring in pig farms	Exceptional biosecurity, low prevalence in imports
**Denmark (1995)** [42–44]	- Mandatory - Surveillance and control - Regular bacteriological and serological testing of whole pig production chain - Risk categorization - Strong biosecurity measures - Surveillance at slaughterhouses	2–3% prevalence of *Salmonella* in breeding and multiplier pigs (based on serology) and 9–15% prevalence in slaughter pigs (based on serology) Low prevalence < 1% in carcass swabs (based on bacteriology)	High compliance with hygiene measures and transparent reporting systems Success but at high cost
**Germany (2002)** [31, 45]	- Mandatory - Surveillance and control - Serological testing of fattening and slaughtered pigs - Biosecurity and vaccination in high-risk areas	Decreasing trend in *Salmonella* prevalence in fattening pigs (based on serology)	Stringent controls in farms and slaughterhouses Minimum success after 20 years
United Kingdom* (2002) [46, 47]	- Mandatory for QA** abattoirs - Surveillance and control - Serological testing of slaughtered pigs	No significant improvement in seroprevalence [48]	Discontinued in 2012
**Ireland (2003)** (SI No. 165/2002 Abattoirs Act 1966: Veterinary Examination Amendment Regulations; SI No. 521/2009 y 522/2009)	- Mandatory - Surveillance and control - Monitoring via on-farm bacteriological sampling (finishers) and serological testing of slaughtered pigs - Biosecurity measures - Focus on high-risk farms	- Decreasing trend in carcass *Salmonella* prevalence (probably due to hygiene practices at slaughter). - Seroprevalence of slaughter pigs has remained stable	Ongoing focus on reducing contamination
**The Netherlands (2005)** [49]	- Mandatory - Surveillance and control - Serological testing of fattening and slaughtered pigs - Biosecurity improvements	Current information on the *Salmonella* status of Dutch herds not available (QA-organizations do not publish results)	Difficulty eliminating persistent strains
**Belgium (2007)** [50–52]	- Voluntary (since 2015) - Maintained for certain quality labels (e.g. Bepork) - Surveillance and control - Serological testing of fattening pigs	No significant improvement in seroprevalence has been observed	Facing ongoing challenges in collaboration with farmers and food industries. Difficulty of ensuring widespread compliance Variations in the effectiveness of biosecurity practices Differences in farm management
**Estonia (2014)** [35, 36]	- Mandatory for high-risk pig herds - Surveillance and control - Targeted serological and bacteriological sampling	Pig prevalence remains high (> 20%) at the farm level and around 3% at the slaughterhouse (based on bacteriology) [53]	Better farm practices and hygiene, though still facing challenges
*Pre-Brexit; ** Quality Assurance			

### What is wrong with the EU national control programs?

Among the NCP for pig salmonellosis initiated by European MS, only a few can be considered successful, often at considerable economic costs. In some cases, such as in the United Kingdom (UK) and Belgium, the programs have been discontinued or reduced to voluntary biosecurity guidance. Various reasons may explain this lack of success, including each country’s specifics—pig census, farm types, production systems and climate. Program implementation also varied, some being voluntary, others compulsory, and enforcement penalties were inconsistent [54]. Countries with strong enforcement, that is, actions to clean up contaminated farms, and penalties, had the most successful *Salmonella* programs.

Where programs were not mandatory nor penalties considered, the farmer’s perception of the problem certainly played an important part [55]. Effective *Salmonella* control requires changes in daily practices, dependent on farmers’ motivation, which in turn may depend on receiving tangible benefits from their actions [56, 57]. However, since porcine salmonellosis is often asymptomatic, direct benefits are unclear, reducing farmer interest.

Technical issues also contributed to the failure of the programs and the frustration of farmers. These issues likely relate to the structure of the pig sector and its impact on the epidemiology of the infection. In addition, the sampling methods employed and the use of imperfect serological tests (see below) to monitor *Salmonella* infection have undoubtedly contributed to the lack of success of these NCP. Understanding the importance of these factors is crucial for developing new and effective control strategies for swine salmonellosis in the future.

#### The structure of the pig sector and the epidemiology of the infection

The pig production system is complex, involving various production periods that may occur on different farms. For the sake of simplicity, it typically begins with sow farms, encompassing gestation, farrowing, and lactation. Following weaning, usually at three to four weeks, piglets move to nurseries until nine to ten weeks. Subsequently, the animals undergo a period of growth and fattening before being sent to slaughter at approximately 20 to 22 weeks of age. Some farms cover the entire production cycle (farrow-to-finish), while others focus on specific stages, such as breeding and nursery, nursery care alone, nursery and fattening (isowean units), or fattening alone. In some systems, batch production is also considered to prevent the spread of diseases.

In recent years, EU pig production has consolidated into large-scale farms [58]. Over 75% of pigs are raised on large commercial farms, that is, over 2,000 production (fattening) pigs [59, 60], with Denmark having the largest average herd size (4,700) and Germany the smallest (1,900). A special case is Spain, with a predominately intensive pig sector. It experienced a two-thirds drop in holdings (128,000) from 1999 to 2013 while the number of pigs per holding quadrupled. From 2014 to 2023, Spain has been the primary contributor to the growth in the EU pig census [60].

One consequence of the intensification process is the emergence of greater specialization among farming operations. An increasing number of farms have prioritized the construction of large fattening units typically situated in locations distant from the breeding-only farms from which their pigs originate. This practice is intended to enhance biosecurity [20]. Control efforts for pig salmonellosis have focused mostly on these fattening units, largely because this is the period immediately preceding slaughter. Therefore, the majority of epidemiological studies on the infection have been conducted during this period [61–69].

However, outcomes have been unsatisfactory due to *Salmonella’s* resilience and its interaction with farm- and animal-related factors such as the type of infrastructure, farm external biosecurity, farm hygiene and disinfection, animal origin, animal management and associated stress, or concomitant infections with other enteric pathogens such as *Lawsonia intracellularis*. Risk factors associated with pig salmonellosis have been extensively described in the literature [70, 71]. The direct consequence of combining a highly versatile pathogen with the multitude of risk factors present on the farm (and at varying levels over the year) is that the presence of *Salmonella* in a herd is typically unpredictable [72]. The occurrence of *Salmonella* varies between and within age groups and pens within herds [63], and its presence is often inconsistent across batches [73].

*Salmonella* infections in fattening pigs may also originate from previous production phases, notably breeding sows, lactating piglets and the nursery, which have been the subject of comparatively little research [74–76]. A 2008 EFSA survey indicated that about 30% of sow holdings were *Salmonella*-positive, with higher prevalences in major pig-producing countries like Spain, the Netherlands, and Denmark [26]. The results of various serological and bacteriological surveys also indicate that the detection of high levels of *Salmonella* in sows is a common occurrence [77–80]. Infected sows and subsequent early infections occurring between birth and weaning are likely to play a pivotal role in the transmission and maintenance of *Salmonella*, increasing the overall likelihood of exposure to the bacteria in further production phases [81, 82].

There is a paucity of comprehensive studies on the prevalence of *Salmonella* in suckling piglets [76], mainly due to challenges in assessing the true infection status. Most research relies on fecal samples, finding low levels of shedding [63, 83–89]. Maternal immunity may influence these results [63, 89–91]. The limited amount of fecal matter typically obtained from rectal swabs, particularly in very young piglets, and the low sensitivity of bacteriology when performed on this matrix [92–94] may also have contributed to the underestimation of the true prevalence of infection in these animals. The prevailing view that weaning- or post-weaning-age pigs would be among the most clinically affected if they had become infected by *Salmonella* [22] has reinforced the perception that the prevalence of *Salmonella* at these ages is very low. In addition, until recently, antibiotics used to control other enteric pathogens in piglets (e.g., *E.coli*) likely masked *Salmonella* detection.

Recent studies on weaning piglets found a 36% *Salmonella* prevalence, with serotypes matching those in sows, suggesting that infected sows are the likely source for exposure and subsequent infection in piglets, which in turn would impact later production stages [78, 95]. It is therefore probable that infected sows are ultimately responsible for infections occurring in the fattening units. Indeed, a risk assessment model indicated sow prevalence as a strong indicator of slaughter pig prevalence [96].

*Salmonella* transmission from sow to piglet mainly occurs via fecal contamination, but other pathways might exist. Evidence of congenital transmission of NTS from dairy cows to newborn calves was proposed in 2016 [97], and it could be happening in swine as well. If proven, new questions will be raised. For instance, whether persistently infected piglets may develop. This type of transmission, if confirmed, would have profound implications for the way this infection should be controlled on pig farms. Regardless of congenital transmission, minimizing sow-piglet transmission is crucial. Strategies to prevent *Salmonella* shedding in sows should be prioritized to control pig salmonellosis in the pre-harvest period.

#### Sampling procedures and the use of imperfect tests

In Europe, many commercial pig farms exceed 2,000 pigs [59, 60]. To accurately assess herd health, sampling must consider herd size. For a herd of 2,000 pigs, a perfect diagnostic test would require sampling between 70 (with 1% expected sero/prevalence) and 321 pigs (with 50% expected sero/prevalence) for a 95% confidence interval and 5% error (Win Episcope; http://www.winepi.net/index.php). A typical fattening farm may market 4,000 to 6,000 pigs annually (between two and three fattening cycles per year), depending on its performance and production systems. Consequently, the number of animals sampled in NCPs often fails to yield precise health estimates.

Additionally, sampled animals (the study population) must represent the entire fattening unit (target population), as a strict all-in/all-out strategy is expected to be followed by those farms engaged in a *Salmonella* control program. Therefore, random selection is crucial to ensure equal probabilities of selection. Some NCPs exhibit significant bias in animal selection, particularly when using carcasses from slaughterhouses, which may not accurately reflect the distribution of pigs on the farm as they have been previously mixed during transportation and lairage. Given the evidence suggesting that *Salmonella* infection distribution in the herd is clustered, with the potential presence of herd subpopulations [98], results obtained from carcasses are likely giving a biased estimate of the health status of the herd. The sampling timing is also critical, as sampling at the beginning of the growing/fattening period may not reflect further changes in sero/prevalence. In contrast, sampling close to slaughter time can provide insights into the potential risk these pigs may pose for slaughter and carcass contamination.

Serological testing is the standard for monitoring *Salmonella* infection [54]. Indirect ELISA tests are quick and cost-effective [99, 100] and can be performed on serum or meat juice samples, targeting major *Salmonella* serotypes affecting pigs [101, 102] However, current serological tests for detecting *Salmonella*-specific antibodies are far from perfect, and their overall diagnostic accuracy is low. Studies show that serological testing often detects only a small fraction (15%) of *Salmonella*-shedding pigs [103], with sensitivities ranging from 59 to 65% depending on the ELISA used [102]. More recent studies employing Bayesian approaches reported ELISA sensitivities as low as 45% [104] In general, the sensitivity of these tests can only be enhanced at the expense of a notable reduction in their specificities [105, 106].

Discrepancies between bacteriological and serological tests arise from various factors, including the timing of infection, serovar diversity [69, 107–109], or even the possibility of seropositive pigs becoming seronegative [63, 110, 111]. A further issue is the overall lack of agreement between serological tests at the individual level. This inconsistency appears to depend on the specific cutoff point recommended for each test [102, 105, 112, 113].

While some researchers find satisfactory herd-level agreement between serology and bacteriology [102], which may be useful for gaining insight into the circulation of *Salmonella* within a farm at a given moment, using serological tests to classify herds could misrepresent their true health status. It has been shown that when categorizing herds on different serological tests, significant discrepancies are observed [106, 113].

Focusing solely on the growing-finishing period for *Salmonella* control may be ineffective when the objective is to reduce the overall prevalence of *Salmonella* and the subsequent infection in humans attributed to pigs. The source of the infection may be in earlier stages of production that are not included in NCPs. This problem may be even more important in countries with intensive pig industries, where fattening units are separate from sow and nursery units. The limited and potentially unrepresentative sampling in many NCPs, coupled with the use of imperfect serological tests, results in biased health status estimates, leading to farmer skepticism and undermining effective on-farm *Salmonella* control measures.

### New approaches for the control of pig-derived salmonellosis

The limited effectiveness and high costs of NCPs for pig salmonellosis, especially in countries with large pig populations [114, 115], highlight the need for reassessment. In the EU, pig salmonellosis is primarily a public health issue rather than an animal health concern. Thus, the main objective of any NCP should be to reduce human salmonellosis incidences linked to pork consumption. However, if consumers and public health authorities benefit from effective control programs while costs fall solely on pig producers without compensation, voluntary farmer participation will likely be challenging. As is the case with other NCPs targeting zoonotic diseases (e.g., brucellosis, tuberculosis), penalties and/or compensation mechanisms should be considered, but given the current high prevalence of infection, costs in major pig-producing countries could be substantial.

Nevertheless, it is vital for farmers to understand the public health implications of salmonellosis in their pigs and their potential role in reducing it before engaging them in on-farm control activities. Once this awareness is established, modifying practices and implementing new ones will become easier [57]. However, given the infection’s widespread nature, it may take time to observe positive outcomes from these efforts. In the meantime, new strategies are necessary to reduce human salmonellosis more rapidly. They should probably be implemented at the interface between the farm and the slaughterhouse.

People become ill after consuming contaminated pork, which often originates from pig carcasses contaminated during slaughter. The primary risk to humans arises from slaughtering pigs with high *Salmonella* concentrations in their feces [116]. It has been shown that a correlation exists between high cecal *Salmonella* loads in pigs and carcass contamination [117]. Asymptomatic *Salmonella*-infected pigs arriving at slaughterhouses are thus the main source of carcass contamination [118–120]. These pigs are particularly prone to *Salmonella* shedding due to pre-slaughter stressors like feed withdrawal, transport, and lairage [121–126]. Consequently, strategies should be developed to prevent or minimize the contamination of slaughterhouses by *Salmonella* from these animals. Such strategies may prove to be a more cost-effective short-term solution than attempting to control the infection on farms. Preferably, these strategies should be applicable regardless of the farmers’ willingness to modify their production practices.

#### The farm-slaughterhouse interface

The relationship between herd *Salmonella* status and pig carcass contamination is well documented [25, 127, 128], but complex. The presence of *Salmonella* at the slaughterhouse may depend on several factors, such as recent pig seroconversion, pre-slaughter stressors, and lairage contamination [129–134], or the presence of *Salmonella* in the mesenteric lymph nodes of infected pigs [135]. Moreover, the implementation of proper slaughter procedures can significantly reduce the risk of contamination [128]. However, preventing carcass contamination cannot rely solely on slaughter activities, especially when *Salmonella* infection prevalence is high [128].

The only baseline study conducted in the EU indicated that, on average, 10% of slaughter pigs are infected, with some countries allowing up to a third of infected pigs into their slaughterhouses [25]. It is expected that a significant number of pigs will shed *Salmonella* while at lairage. A recent study in Spain found that 27.3% of slaughter pigs were shedding *Salmonella* [136]. Lairage contamination may result in new infections and an increase in environmental contamination as this area cannot be adequately cleaned and disinfected during the day [137]. From there, the contamination can spread to the slaughter room, clean room, and even into the chillers, via vectors, fomites, or airborne transmission [138].

**Fig. 2:**
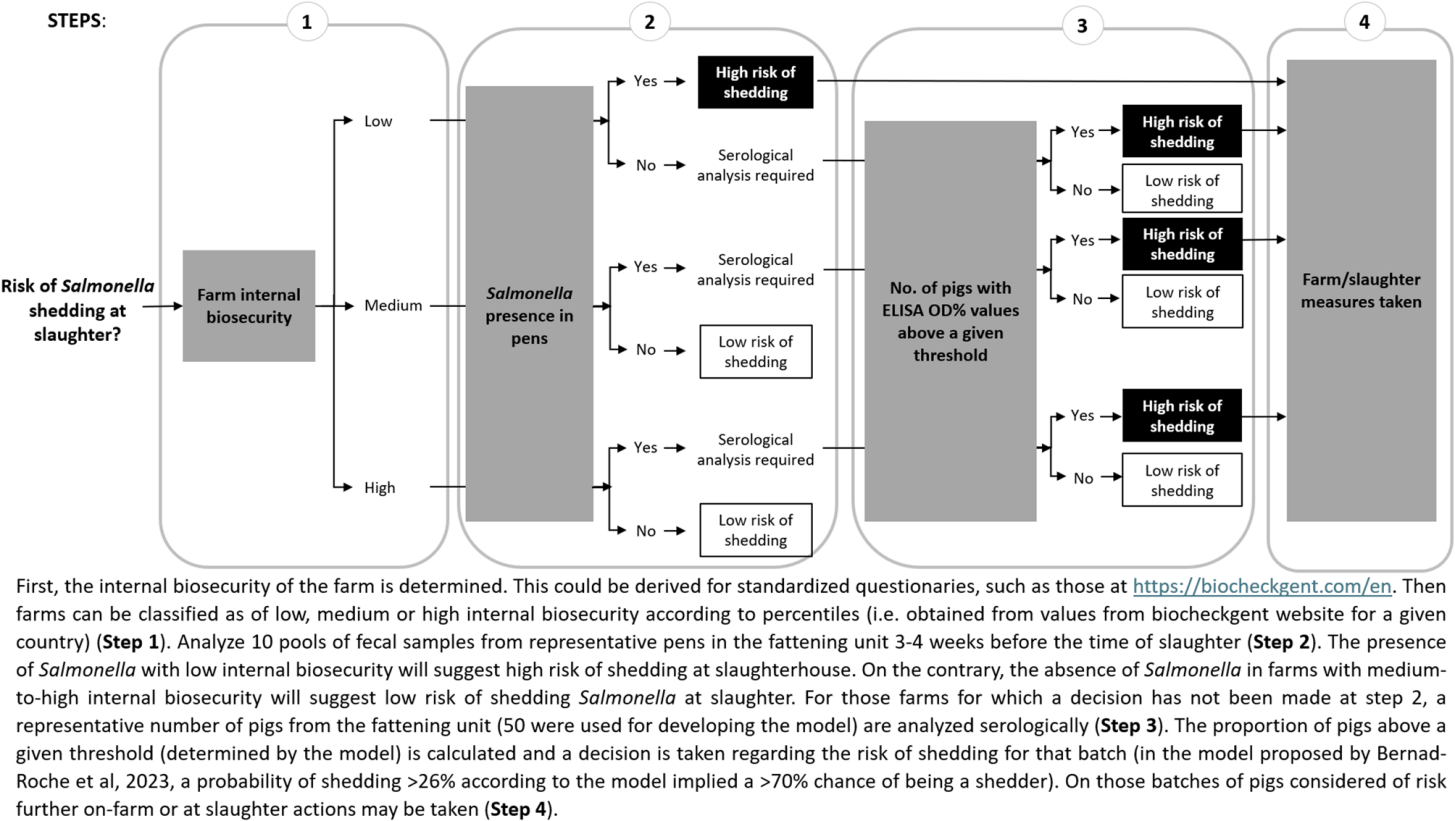
Decision tree to assess the risk of shedding *Salmonella* at the slaughterhouse for a given fattening unit based on three variables, the farm internal biosecurity score, the presence of *Salmonella* in 10 representative pens and the serological results of 50 representative fattening pigs

Although serology does not show a strong correlation with on-farm *Salmonella* shedding, this relationship becomes more evident when comparing serological results with shedding at the slaughterhouse [69, 135]. At the farm-slaughter interface, on-farm serology, along with two farmer-independent variables—the farm’s internal biosecurity score and the prevalence of *Salmonella* in pens—has proven reliable for predicting batches of pigs with a high likelihood of shedding *Salmonella* during lairage [139]. Interestingly, serological data were not always necessary in the model by Bernad-Roche et al. Pigs from farms with low internal biosecurity and *Salmonella* in the pens were considered at high risk of shedding, regardless of serological results. Conversely, pigs from farms with good biosecurity and no detected *Salmonella* should be viewed as low risk, simplifying the approach. Consequently, a decision tree for reducing shedding risk at the slaughterhouse could be developed based on this model (Fig. 2). If a significant number of pigs from a specific batch are predicted to shed *Salmonella* prior to slaughter, both on-farm and on-slaughterhouse preventive measures can be identified to reduce the risk of slaughter contamination.

#### On-farm measures

In the final 2–3 weeks of fattening, measures for high-risk batches should aim at reducing *Salmonella* shedding both on the farm and at the slaughterhouse. Lowering shedding on the farm will reduce the likelihood of new infections, thus reducing contamination during transport and lairage. It will also lower infection pressure at the slaughterhouse.

At this stage, when only a few weeks or days remain before slaughter, the interventions must be capable of rapidly eliminating bacteria while remaining effective until slaughter and safe for consumers (no withdrawal period should be required). It can be reasonably assumed that the ingestion of products designed to reduce the burden of *Salmonella* in the intestinal tract is the only on-farm intervention that can be expected at this stage.

The use of coarse meal in pig feed has been associated with a decrease in *Salmonella* prevalence when compared to diets comprising finely ground or pelleted feed. The addition of coarse meal increases gastric retention time and encourages fermentation, leading to higher levels of organic acids (e.g., lactic acid), lower pH, and a richer anaerobic microbiota. These gut improvements have been shown to significantly reduce *Salmonella* survival during gastric transit and correlate with lower *Salmonella* shedding at slaughter, suggesting coarse meal could serve as a potential strategy for integrated *Salmonella* control [140].

However, given that pigs are typically subjected to a fasting period of 12 to 24 h prior to slaughter, a critical period for bacterial shedding, drinking water should probably be also considered a primary vehicle for most products. Water will be available to the animals until they are loaded onto the truck and, subsequently, in the lairage area. Prebiotics, probiotics, different types of organic acids, and more recently, postbiotics, parabiotics, and bacteriophages, have shown promising antibacterial properties and could be administrated through water (see below).

#### Prebiotics and probiotics

Prebiotics and probiotics can enhance animal health by influencing gut microbiota by stimulating beneficial bacteria (prebiotics) or directly increasing their populations (probiotics) [141]. This leads to a healthier digestive tract and immune system via mechanisms such as competitive exclusion and immune modulation, as well as the production of useful metabolites, enzymes, or bacteriocins [142].

The efficacy of probiotics is contingent upon the interaction between the host (e.g. age, health, immune status) and the probiotic microorganism (e.g. strain, single or combination of several strains, dose) [143]. The impact of probiotics on *Salmonella* control has been primarily observed in piglets (suckling or postweaning), where gut flora is still in development [144–146]. In finishing pigs, where the microbiota is more stable [147], the treatment period may need to be much longer. Moreover, not all probiotics have proven effective against *Salmonella* [148], and safety remains a concern [143]. Strain selection must be carefully studied, with each probiotic requiring thorough safety and risk assessments [149]. If drinking water is used for administration, factors such as chlorination should be considered to prevent the reduction of probiotic doses.

Prebiotics should also require long periods of treatment to observe a positive effect. Some studies on piglets have shown reductions in *Enterobacteriaceae* and *Salmonella* counts after lengthy treatment periods (≈ 4 weeks) [150, 151]. In older pigs, there is only one study showing that the administration of prebiotics (a β-galacto mannan oligosaccharide) resulted in a reduction of *Salmonella* shedding and infection at the time of slaughter, but its effect was observed after two months of treatment [152].

Therefore, it remains uncertain whether probiotics and prebiotics could prove beneficial in reducing shedding during the final days of fattening. Further studies are needed to assess whether they can be used to reduce *Salmonella* shedding prior to slaughter in such a short time.

#### Postbiotics and parabiotics

The fields of postbiotics and parabiotics (PP) represent a novel area of research within the disciplines of animal nutrition, preventive veterinary medicine, and production [153]. Postbiotics are defined as the metabolic products secreted by probiotics, including enzymes, proteins, and peptides. In contrast, parabiotics are inactivated microbial cells containing components such as peptidoglycans, teichoic acids, surface proteins, or crude cell extracts [154]. PP are regarded as a safer alternative to probiotics, as they do not pose the same biological risks, such as bacterial translocation from the gut lumen to the bloodstream or the transfer of antibiotic resistance [155].

The available evidence suggests that PP can enhance animal performance and reduce *Enterobacteriaceae* counts and diarrhea [156]. However, most of trials have been conducted on young piglets and have lasted four to five weeks, which limits the applicability of these findings to pigs near slaughter. Despite the growing body of evidence supporting the health benefits of PP, further research is needed to elucidate their mechanisms of action, develop *Salmonella*-targeted PP, and establish international definitions for their regulation [157].

#### Organic acids

The addition of organic acids (OA) in feed or drinking water represents one of the most extensively researched strategies for the control of swine salmonellosis not only because of its direct antimicrobial activity on potential pathogens present in feed or water, but also because of its effects on the gastrointestinal tract of the animals. The bactericidal action of these acids is due to their ability to cross the cell membrane, dissociate inside where the pH is more alkaline, and acidify the cell cytoplasm, affecting protein and DNA synthesis and causing cell death [158]. In addition to this effect, the mechanisms of action of these acids in the gastrointestinal tract are numerous. Firstly, they lower the pH, mainly in the anterior sections of the gastrointestinal tract, as acids are normally absorbed along the small intestine, thus reinforcing the stomach as an entry barrier for *Salmonella*. Secondly, they stimulate the growth of epithelial cells [159]. It has been shown that certain acids, mainly butyric, caproic and caprylic acids, can also reduce the expression of *Salmonella* pathogenicity genes, thus limiting their capacity to colonize the intestinal epithelium of pigs [160, 161]. Nevertheless, some studies have suggested that some short-chain fatty acids may also induce the expression of invasion genes in *Salmonella* [162, 163].

While the early use of OA faced several challenges, such as the corrosive effects on watering pipes, poor palatability, and difficulty reaching the posterior sections of the gastrointestinal tract (ileum, caecum, colon), where *Salmonella* typically colonizes, these issues have been effectively overcome through the microencapsulation of OA [159, 164].

Nevertheless, the efficacy of OA for the control of *Salmonella* in pigs has been variable, contingent upon factors such as the different study designs (e.g., piglets vs. fattening pigs, natural vs. experimental infection, different administration periods), the type of OA used or the dose applied [165–170]. In addition, a potential adverse effect is the development of acid resistance, which would reduce the efficacy of these agents [171].

In general, beneficial results have been observed after the administration of OA for at least four weeks [167, 172–175], and it appears to be a cost-effective measure to reduce *Salmonella* prevalence along the pork production chain [176, 177]. However, the need for prolonged treatment periods raises doubts about their suitability when applied to finishing pigs prior to slaughter.

A particularly interesting type of OA is that which has undergone esterification. They are short- and medium-chain fatty acids combined with glycerol and have shown enhanced antimicrobial activity against Gram-negative bacteria in both in vitro and in vivo settings [178]. Esterified OA have shown additional advantages, including reduced pH dependence and enzymatic breakdown susceptibility, which allows for activity across the entire gastrointestinal tract [179]. Furthermore, they possess an amphipathic structure that allows them to be soluble in water without altering the pH. Additionally, they are odorless and non-corrosive, which prevents any off-flavors in the water that might deter the pigs from drinking it.

A recent study using an esterified form of formic acid showed that the inclusion of 10 kg/1000L of this acid into the farm water supply for five days prior to slaughter effectively reduced the shedding of *Salmonella* by 82% [180]. The treatment also resulted in a significant reduction in the *Salmonella* loads in pigs that continued to shed the bacteria. The same dosage was observed to reduce the proportion of shedders by up to 63% when the treatment was applied exclusively in the drinking water of the lairage area [136]. These results indicate that this esterified form of formic acid may be a promising product within an overall strategy to minimize *Salmonella* shedding at slaughter.

In general, OA, and particularly those that could be easily blended with drinking water, appear to be a feasible strategy to reduce the shedding of slaughter pigs. The use of OA is safe and could be administered even during the stay of the pigs in the lairage area, thereby increasing the likelihood of timely elimination of the bacteria from the pigs’ gut.

#### Bacteriophages

The use of bacteriophages (phages) offers a promising strategy for the reduction of *Salmonella* loads within the intestinal tract of finishing pigs. Phages exclusively infect bacteria, thus they are not harmful to animal cells or consumers [181]. They can lyse multidrug-resistant (MDR) strains [182] and their effects are observed quickly [183, 184]. Both in-feed and in-water delivery methods are effective as phages multiply in the gastrointestinal tract while bacteria are present [185]. While a minimum bacterial presence is needed for phage propagation [186], this condition is likely to be met in most of the target pigs.

*Salmonella* phages are abundant in pig slurry [187] and can be easily obtained and selected for use [188]. Each year, new phages are being characterized for potential use in commercial farms [189–191]. However, there are several biological and technical obstacles that must be overcome before phages can be employed to treat *Salmonella* in finishing pigs. First, only virulent (non-lysogenic) phages should be selected to ensure bacterial elimination [192]. Phages are also highly specific, often targeting only a particular species, serotype, or subset of strains [193]. Therefore, it is of paramount importance to select the appropriate phage for the target *Salmonella* serotype. Knowledge of the most prevalent serotypes on the farm is needed, but focusing on zoonotic serotypes like Typhimurium, its monophasic variant, and Derby may prove effective. In addition, the use of phage cocktails can help broaden the host range [193].

Phages are susceptible to a range of external factors, including temperature, acidity, salinity, and ions [194]. Therefore, delivery methods must ensure phage survival. Water or feed are anticipated as vehicles for the on-farm administration of phages, and factors such as water composition, chlorination, feed pelleting, and stomach acidity can reduce their survival [182]. Solutions to this challenge include the use of phages in buffer solutions, encapsulation, and freeze- or spray-drying [182, 195, 196]. Ensuring phage stability remains a key challenge for the industry [197].

A potential risk associated with the use of phages is the emergence of phage-resistant *Salmonella* variants, which typically occurs through spontaneous mutations [198]. Experimental studies show that resistance can develop within hours after exposure to a single phage, primarily through mutations in lipopolysaccharide (LPS) biosynthetic genes [199, 200]. This can be also mitigated by the use of phage cocktails [201–203]. Interestingly, the development of phage resistance can render bacteria more susceptible to environmental factors and antibiotics [200].

The use of phages has significantly reduced *Salmonella* colonization and shedding in post-weaned pigs [183–185, 204, 205]. It is expected that similar results would be observed in older animals, thus making them a suitable intervention for the treatment of pigs at high risk of shedding *Salmonella* at the time of slaughter. Although a comprehensive regulatory framework for phage therapy in veterinary medicine is still lacking in most countries, progress has been made. Phages are not yet authorized in the EU, but the European Medicines Agency (EMA) has recently issued guidelines on the quality, safety, and efficacy of veterinary medicinal products for phage therapy [206]. These guidelines provide clear regulatory, technical, and scientific requirements for phage-based veterinary medicines.

#### Additional on-farm interventions

While the implementation of these strategies may assist in reducing the shedding of *Salmonella* at the slaughterhouse and subsequent contamination of the pig carcasses, the original sources of *Salmonella* infection will remain unaddressed, contributing to the sustained high *Salmonella* prevalence in many farms. However, the routine sampling of a representative number of pens and animals on the farm and the identification of batches of high risk of *Salmonella* shedding would provide accurate information to properly identify risk farms. This information could be used to prompt further investigations into the sources of infection in these farms and the implementation of additional, more general, on-farm interventions.

Most activities on pig farms should focus on preventing new *Salmonella* infections. This objective can be accomplished through three fundamental interventions: the sanitary control of feed and breeding animals, as well as the enhancement of biosecurity measures.

Feed contamination is recognized as a significant route for the introduction of *Salmonella* into pig farms. It has been estimated to account for up to 14.2% of the total infection risk [207]. All Member States (MS) runs a rather equal risk because the high risk feed ingredients (vegetable protein, in particular soybeans- and rapeseed meal) are equally used in all MS, and when studied found to be frequently contaminated. The risk may be higher in countries using animal derived proteins. The relative importance of the risk of introducing *Salmonella* by feed into farms is higher in low prevalence countries where other sources largely are minimized. Those countries generally also apply special measures to minimize the risk for introducing *Salmonella* by contaminated feed. In the absence of such measures, contaminated feed will jeopardize efforts to improve the *Salmonella* status also in high prevalence countries [208–210].

Swine producer should ensure that feed mills are implementing Hazard Analysis and Critical Control Points (HACCP)-based program according to EU Regulation (183/2005 EC), which in detail presents the elements involved, and the role of the competent authority and the feed operator. Control measures include heat treatment (e.g., pelleting at high temperatures), the use of organic acids or other antimicrobial additives, protocols for hygiene in feed production facilities, and regular microbiological monitoring of both raw materials and finished products and, most important, that corrective interventions are undertaken when *Salmonella* is isolated. Furthermore, the sourcing of certified *Salmonella*-free ingredients is emphasized [42]. Studies have shown that when implemented effectively, HACCP systems can be successful in reducing and even eliminating *Salmonella* contamination in feed and feed ingredients, consequently leading to a decline in the number of infected farms [209, 211]. The production of *Salmonella* safe feed requires that all feed business operators have implement the EU regulation and can document that prescribed procedures are maintained. However, the efficacy of this mandate largely depends on enforcement at the national level, and compliance may vary among feed producers, particularly in regions with limited official control. Consequently, farmers may rely on feed mills that choose to implement rigorous HACCP-based programs [212].

Effective feed management on pig farms is also crucial to prevent *Salmonella* recontamination, particularly when feed is mixed or processed on-site. Even when employing ingredients that have been certified as safe, inadequate on-farm practices, including improper storage, a lack of pest control, or insufficient cleaning of mixers and silos, can jeopardize the safety of the feed. Farms that produce their own feed should use heat-treated components and refrain from long storage periods. Sealed storage, rodent control, equipment hygiene, and strict biosecurity during handling are all key to reducing the risk of infection [211].

Breeding sows also play an important role in the transmission of *Salmonella* to piglets. Therefore, in breeding farms, the efforts should be focused on minimizing the shedding of *Salmonella* in sows. As with finishing pigs, the routine administration to the sows’ feed or drinking water of some organic acids or phages with a rapid bacterial killing effect during the days prior to farrowing could be an effective strategy to decrease *Salmonella* contamination in maternal crates.

*Salmonella* vaccination has been an optimal strategy for the eradication of *Salmonella* in laying hens and to prevent egg contamination [213]. The same approach could be used in swine to limit animal, environmental, and food contamination [79]. Sow vaccination prior to farrowing to boost passive immunity of the suckling piglets [214], which could be combined with piglet vaccination during the nursery period to induce its own immune response to *Salmonella*, would help to reduce *Salmonella* infection and shedding in this initial period of the pig production [215].

Nonetheless, many factors, such as the intracellular mode of *Salmonella* infection, its antigenic diversity and the lack of vaccine cross protection, the high prevalence of infection even from the early life of the animals, and the persistence of the bacteria in the environment, challenge the efficacy of vaccination [215]. A meta-analysis study showed that, irrespective of the type of vaccine used (attenuated or inactivated), this strategy was effective in reducing the number of *Salmonella*-positive samples on the farm, therefore contributing to the reduction of within-farm transmission, but its overall efficacy was limited (< 30%) [216]. Vaccination should therefore be considered as another intervention that could be implemented along with other on-farm measures, but not to rely solely on it. In addition, depending on the vaccine strategy used (e.g. only vaccination of sows, vaccination of piglets or growers), vaccines should be neither used in farms undergoing routine serological monitoring because of their interference with test results [215].

Biosecurity, and particularly internal biosecurity, has been positively associated with reduced *Salmonella* shedding at slaughter [139]. Internal biosecurity is described as those measures intended to prevent the within-herd spread of pathogens as opposed to external biosecurity, which includes measures to avoid the introduction of pathogens from outside the farm [217, 218]. Thus, implementing and maintaining activities such as disease containment, strict hygiene protocols, and proper use of working lines and manure handling, should contribute to a reduction of *Salmonella* circulation within the farm. However, internal and external biosecurity are highly correlated and should be treated as a common strategy if effectiveness is to be maximized [219].

Cleaning and disinfection (C&D) are critical activities to prevent bacteria from remaining in the facilities and the subsequent infection of new animals entering them [220]. *Salmonella* is known for its ability to persist in the environment [221], which is enhanced by the production of biofilms. Consequently, many cleaning and disinfection (C&D) protocols may prove inadequate for eradicating the bacteria [222]. Phages may serve as a supplementary measure alongside C&D protocols for farm facilities, given their capacity to produce lytic compounds and enzymes that disrupt biofilms [223, 224]. The use of autophages, that is, phages isolated from the same farm where the target bacterium has been isolated [225] appears to be a promising strategy to eradicate recalcitrant *Salmonella* strains in farms [226].

#### Interventions at the slaughterhouse

While on-farm *Salmonella* infection represents a significant source of slaughterhouse environmental contamination, the potential for *Salmonella* contamination exists at any stage of pork production. The ultimate carcass status depends upon the slaughtering conditions [118], thereby underscoring the pivotal role of the slaughterhouse.

Slaughters are subject to considerable pressure due to the high prevalence of *Salmonella*-infected pigs received. Consequently, rigorous measures must be implemented along the slaughter line to prevent contamination at any stage. The available evidence indicates that the failure to implement effective control measures at critical points in the slaughter process, such as scalding, dehairing, singeing, evisceration, and facility cleaning, is associated with an increased risk of carcass contamination. Reviews of the efficacy of different slaughter interventions are available elsewhere [227, 228]. Despite the efforts of many slaughterhouses, contamination of pig carcasses persists [6, 118].

One of the primary sources of *Salmonella* contamination in the slaughterhouse is the lairage area. During lairage, stressed infected pigs are more likely to shed *Salmonella*, thereby facilitating its transmission to other pigs within hours [129]. Implementing measures such as reducing lairage time and cleaning between batches may prove an effective means of mitigating the risk of carcass contamination [128, 129, 137]. However, the practical feasibility of such measures may present a significant challenge. Logistic slaughter of *Salmonella*-positive batches is another proposed intervention, but its efficacy is still a matter of debate [228]. The effectiveness of logistic slaughter could be enhanced by segregating high-risk pig batches before delivery [137]. Furthermore, holding these pigs separately and providing water treated with esterified organic acids may reduce *Salmonella* shedding before slaughter [136].

Implementing more straightforward slaughter interventions to approach a zero policy for *Salmonella*-positive carcasses is also a possibility. Methods include physical and chemical decontamination treatments on carcasses, such as chlorine, electrolyzed oxidizing water, and organic acids, which have proven effective [229–232]. However, these can result in side effects such as the proliferation of acid-resistant bacteria [233]. In addition, chlorine-based disinfectants pose safety concerns [234] and they are not widely accepted by European consumers [235]. Physical techniques such as irradiation or pulsed-light UV have also been shown to significantly reduce bacterial counts, but they raise consumer concerns [236, 237]. Bacteriophage cocktails also offer a promising avenue for decontamination, though more research is needed before they can be recommended for widespread use [238]. Furthermore, although these methods are regarded as cost-effective for reducing microbiological contamination on pig carcasses [239–241], their impact should not be overstated. Overreliance may lead to a relaxation of hygiene practices [233]. Therefore, they should be part of a comprehensive food safety system [242]. In light of these concerns, the EU has adopted a cautious stance, permitting only water for carcass decontamination (Regulation (EC) No 853/2004). In contrast, the US allows the use of alternative treatments, including organic acids [243].

Until new interventions are approved, slaughterhouses must continue implementing strict measures along the slaughter line in order to effectively manage the high number of infected pigs that they receive. Accurate knowledge of the *Salmonella* status of incoming pigs would greatly improve the efficiency of these control measures, particularly at the lairage stage, helping to reduce environmental contamination during slaughter.

### Future directions and conclusions

Previous attempts to reduce human salmonellosis in the EU through NCPs targeting pig salmonellosis have largely been unsuccessful, likely due to the difficulty in lowering *Salmonella* infection rates on pig farms. The focus of these programs has primarily been on fattening pigs and slaughterhouses, while earlier stages in the pig production chain, where the infection is also prevalent, have been neglected.

The role of infected sows in the transmission of *Salmonella* to piglets has been the subject of little research. A recent study suggests *Salmonella* could be transmitted congenitally in mammals such as cattle. However, no studies have been conducted on swine in this regard, an animal species that exhibits higher infection rates than cattle. Furthermore, it was previously assumed that infected suckling piglets would become sick, but recent evidence shows they can appear healthy while shedding *Salmonella*, thus spreading it to the nursery. This issue is further aggravated by the ban on antimicrobials like colistin [244], an antibiotic previously used to control gram-negative bacteria. These findings underscore the need for effective salmonellosis control measures to be initiated at the sow farm.

The rapid growth of the pig industry, with more large-scale farms and concentrated production, must be considered when designing effective monitoring programs. Sampling procedures should better reflect the herd’s true *Salmonella* status to foster trust among farmers. Without it, the implementation of new on-farm biosecurity measures will be challenging. The diagnostic accuracy of serological tests is constrained by the nature of this infection, rendering results difficult to interpret. Furthermore, vaccination is only effective in conjunction with strict biosecurity measures.

If there is a real commitment to reducing human salmonellosis linked to pork, there are different strategies that could be used at farm level. It may also be possible to predict the risk of *Salmonella* shedding before pigs arrive at the slaughterhouse. Batches identified as high-risk could be treated on-farm with in-water additives in the days preceding slaughter, in order to reduce shedding and environmental contamination during processing. This approach is likely to be economically feasible, provided that the public health benefit of reducing human salmonellosis is properly recognized. At present, OA represent the most effective short-term treatment option, but alternative methods, such as bacteriophages, may soon emerge. Furthermore, routine on-farm sampling of a representative number of finishing pigs and pens could assist in a more accurate classification of farms according to their *Salmonella* risk, thereby enabling slaughterhouses to optimize slaughter procedures and targeted interventions.

## Data Availability

No datasets were generated or analysed during the current study.

## Abbreviations

C&D: Cleaning and disinfection
DNA: Deoxyribonucleic acid
ECDC: European centre for disease prevention and control
EFSA: European food safety Authority
ELISA: Enzyme-linked immunosorbent assay
EMA: European medicines agency
EU: European union
QA: Quality assurance
QS: Qualität und sicherheit
LPS: Lipopolysaccharide
MS: Member state
NCPs: National control programs
NTS: Non-typhoidal salmonella
OA: Organic acids
PP: Postbiotics and parabiotics
UK: United Kingdom
US: United States
WHO: World health organization
